# Algorithmic tools for tripartite data analysis

**DOI:** 10.1186/1471-2105-15-S10-P32

**Published:** 2014-09-29

**Authors:** Charles A Phillips, Erich J Baker, Elissa J Chesler, Michael A Langston

**Affiliations:** 1 Department of Electrical Engineering and Computer Science, University of Tennessee, Knoxville, TN 37996, USA; 2 Bioinformatics Program, School of Engineering and Computer Science, Baylor University, Waco, TX 76798, USA; 3 The Jackson Laboratory, Bar Harbor, ME 04609, USA

## Background

Bipartite graphs have many applications. Examples include the modeling of gene-disease associations, substrate-enzyme relationships and protein-protein interactions. Numerous algorithms have been proposed to extract dense subgraphs from bipartite graphs.

## Materials and methods

In this work, tripartite graphs are considered. Applications include comparing two sets of many gene-many disease associations. An algorithm is described that finds a maximum triclique in such a graph. It employs a branching strategy inspired by maximum clique algorithms for general graphs. A binary search tree is used, in which branch nodes in the tree represent vertices in the tripartite graph, and in which branching decisions are based on whether a vertex is in or out of a maximum triclique. A reduction rule is also introduced to filter out irrelevant vertices. This algorithm was developed in the context of GeneWeaver, an online system for the integration of functional genomics experimental results. In this system triclique extraction will enable fast transitive association of diseases based on the similarity of gene-disease associations from many experiments. Computational experience with huge volumes of experimental data is described.

